# Genetic Associations Between IL-6 and the Development of Autoimmune Arthritis Are Gender-Specific

**DOI:** 10.3389/fimmu.2021.707617

**Published:** 2021-09-03

**Authors:** Jianqiao Hong, Zihao Qu, Xiaoxiao Ji, Congsun Li, Geng Zhang, Ciliang Jin, Jie Wang, Yongxing Zhang, Yue Shen, Jiahong Meng, Chenhe Zhou, Chaohua Fang, Wei Wang, Shigui Yan

**Affiliations:** ^1^Department of Orthopedic Surgery, The Second Affiliated Hospital, Zhejiang University School of Medicine, Hangzhou, China; ^2^Orthopedic Research Institute of Zhejiang University, Hangzhou, China; ^3^Key Laboratory of Motor System Disease Research and Precision Therapy of Zhejiang Province, The Second Affiliated Hospital, Zhejiang University, Hangzhou, China; ^4^State Key Laboratory for Diagnosis and Treatment of Infectious Diseases, National Clinical Research Center for Infectious Diseases, Collaborative Innovation Center for Diagnosis and Treatment of Infectious Diseases, Department of Infectious Diseases, The First Affiliated Hospital, College of Medicine, Zhejiang University, Hangzhou, China; ^5^Joint Surgery, Ningbo 6th Hospital, Ningbo, China

**Keywords:** Mendelian randomization, IL6, sIL6R, autoimmune arthritis, ADAR

## Abstract

**Objectives:**

To find out the genetic association between IL6 and autoimmune arthritis.

**Methods:**

We performed a two-sample Mendelian randomization (MR) study using multiple genome-wide association studies (GWAS) datasets. Furthermore, a sex-stratified MR study was performed to identify sexual dimorphism in the association between IL6 and autoimmune arthritis. Then, LocusZoom plots were displayed based on the IL6R gene region to present evidence of genetic colocalization between diseases.

**Results:**

The MR result denoted a genetic association between the increased level of IL-6 signaling and risk of RA (β=0.325, 95%CI 0.088, 0.561, p=7.08E-03) and AS (β=1.240, 95%CI 0.495, 1.980, p=1.1E-03). Accordingly, sIL6R was found to have negatively correlation with the onset of RA (β=-0.020, 95%CI -0.0320, -0.008, p=1.18E-03) and AS (β=-0.125, 95%CI -0.177, -0.073, p=2.29E-06). However, no genetic association between IL6/sIL6R and PsA was detected. The gender-stratified MR analysis showed that IL6 was associated with AS in the male population, with RA in the female population, and with PsA in the male population. Additionally, ADAR, a gene identified by a sensitive test, could be the reason for the nonsignificant association between IL6 and PsA in a pooled population.

**Conclusion:**

Our findings showed that the overactive IL6 signal pathway led to autoimmune arthritis, especially in RA and AS. Sexual difference was also observed in IL6-intermediate susceptibility to autoimmune arthritis.

## Highlights

1. Genetic association was identified between IL6-signaling and autoimmune arthritis.
2. IL6 was correlated with male AS, male PsA, and female RA development in a sex-dependent manner.

## Introduction

Interleukin-6 (IL-6), as a proinflammatory cytokine, plays major roles in the inflammation process and the development of the immune system (1, 2). However, abnormal immune response could lead to autoimmune arthritis including rheumatoid arthritis (RA), ankylosing spondylitis (AS), and psoriatic arthritis (PsA), which places a great burden on patients by causing joint destruction and even ending up with disability (3, 4). As previously reported, elevating levels of IL-6 have been detected in pathological sites among RA, AS, and PsA patients, and the serum level of IL-6 is associated with the autoimmune arthritis pathogenesis (5–7). Hence, IL-6 blockade seems to be a therapeutic target for alleviating the process of autoimmune arthritis. Anti-IL-6 signaling drugs such as tocilizumab and siltuximab have been approved for RA and systemic juvenile idiopathic arthritis therapy by the Food and Drug Administration (FDA) (4). However, treating AS or PsA with anti-IL-6 therapy could only relatively alleviate the progression of diseases in some reports (8, 9).

It is known that genetic susceptibility accounts for 30% in terms of the risk of autoimmune disease (10). By using information from genome-wide association studies (GWAS), Mendelian randomization (MR), a newly introduced approach, has the potential to evaluate the causal relationship between exposures and phenotype (11, 12). Briefly, MR uses genetic variants as the instrumental variable to interpret a different outcome without bias caused by reverse causation or confounding factors (13, 14). Previous MR studies have shown that the genetic level of serum sIL6R is negatively associated with the risk of developing RA (15). Li and his colleges found that the IL-6 gene -174G/C variant is associated with the risk of RA using MR meta-analysis (16). In order to evaluate the casual association between IL-6 and other autoimmune arthritis, large-scale MR study should be carried out to verify the exact correlation and find out potential therapeutic strategy.

In this study, up-to-date GWAS level summary data for IL-6 signaling and circulating sIL6R were utilized in the MR analysis. The results presented a genetic association between IL6 and different types of autoimmune arthritis. Additionally, we further discovered the underlying sexual dimorphism in these autoimmune diseases based on the MR results.

## Method

### Genetic Instrumental Variables

Two sets of IL6-related genetic instruments were based on recent MR reports. Genetic variants that represented for IL6 signaling were obtained from a large-scale GWAS meta-analysis assessing chronic inflammation, which included 204,402 European individuals (17). Based on prior MR reports from Kappelmann et al. and Georgakis et al. (18, 19), who investigated the association between IL6 signaling on specific depressive symptom outcomes and cardiovascular outcomes, respectively, IL6 signaling-related SNPs were used in our study (**Table S1**). According to Kappelmann et al.’s definition, “IL6 signaling” referred to IL-6R genetic instruments and weighted by the level of CRP.

Plasma sIL6R is a decoy receptor that is able to suppress IL6 signaling by forming an inhibitory complex with sgp130 (20). To further identify the role of IL6 signaling in autoimmune arthritis, genetic IV SNPs associated with the level of sIL6R were generated from a European ancestry GWAS (n=3,301) (21). A total of 34 SNPs utilized in this study were based on Rosa et al.’s study (**Table S1**) (15). Linkage disequilibrium levels of SNPs chosen above from two datasets were tested using the LDmatrix Tool (https://ldlink.nci.nih.gov/?tab=ldmatrix, CEU; r2 < 0.1). When IV SNPs could not be found in autoimmune arthritis summary statistics, the LDproxy Tool was used to identify potential proxy SNPs (CEU; r2>0.8).

### Summary Statistics for Autoimmune Arthritis

Publicly available meta-analysis datasets for autoimmune arthritis (RA, AS, PsA) were derived from some different studies. GWAS summary statistics of 14,361 RA cases and 43,923 controls of European ancestry were obtained from a large-scale GWAS (22). The summary of the GWAS statistic for AS and PsA was obtained from FinnGen, a large-scale project combining genotype data from Finnish biobanks and digital health record data from Finnish health registries. We selected datasets containing 541 AS cases, 74,589 controls and 562 PsA cases, and 93,959 controls of Finnish ancestry. Another set of autoimmune arthritis meta-analysis summary statistic was acquired from UK Biobank (UKBB) for replication study, encompassing 361,141 of European ancestry with 4,017 RA cases, 3,154 AS cases, and 712 PsA cases.

Moreover, summary GWAS results of these three types of autoimmune arthritis in different genders were extracted from UKBB (with 194,174 female and 167,020 male) for further sex-stratified MR analysis to avoid potential sexual bias, due to the fact that gender differences had been widely reported in autoimmune diseases.

### Statistical Analysis

To evaluate casual effect, an MR study was carried out with four modes: MR-Egger mode, weighted median mode, and inverse variance-weighted (IVW) mode with the “TwoSampleMR” R-package (23). With the minimum weighted average variance, the IVW method was mainly selected for analysis (24). Because the MR analysis is based on the hypothesis that genetic exposure influences the outcome directly, it is not suitable to perform the analysis in the presence of pleiotropy. Thus, we conducted several approaches to detect potential pleiotropy (25), Firstly, MR-Egger regression was performed to capture the horizontal pleiotropy (26). Additionally, we applied MR pleiotropy residual sum and outlier test (MR-PRESSO) to detect potential horizontal pleiotropy using the MR-PRESSO global test. For any detected pleiotropic SNP, the MR-PRESSO outlier test was performed to remove these SNPs and rectify the horizontal pleiotropy (27).

Heterogeneity test was performed using Cochran’s Q statistics analysis to identify whether the MR results were biased by the heterogenic factors. Leave-one-out sensitivity analysis was performed to identify whether the estimate was driven by an SNP. These two methods were also applied through the “TwoSampleMR” R-package. The estimated effect represented the log odds ratio (OR) between IL6R and autoimmune arthritis risk. All the analyses with P<0.05 were considered statistically significant.

## Results

**Figure 1:**
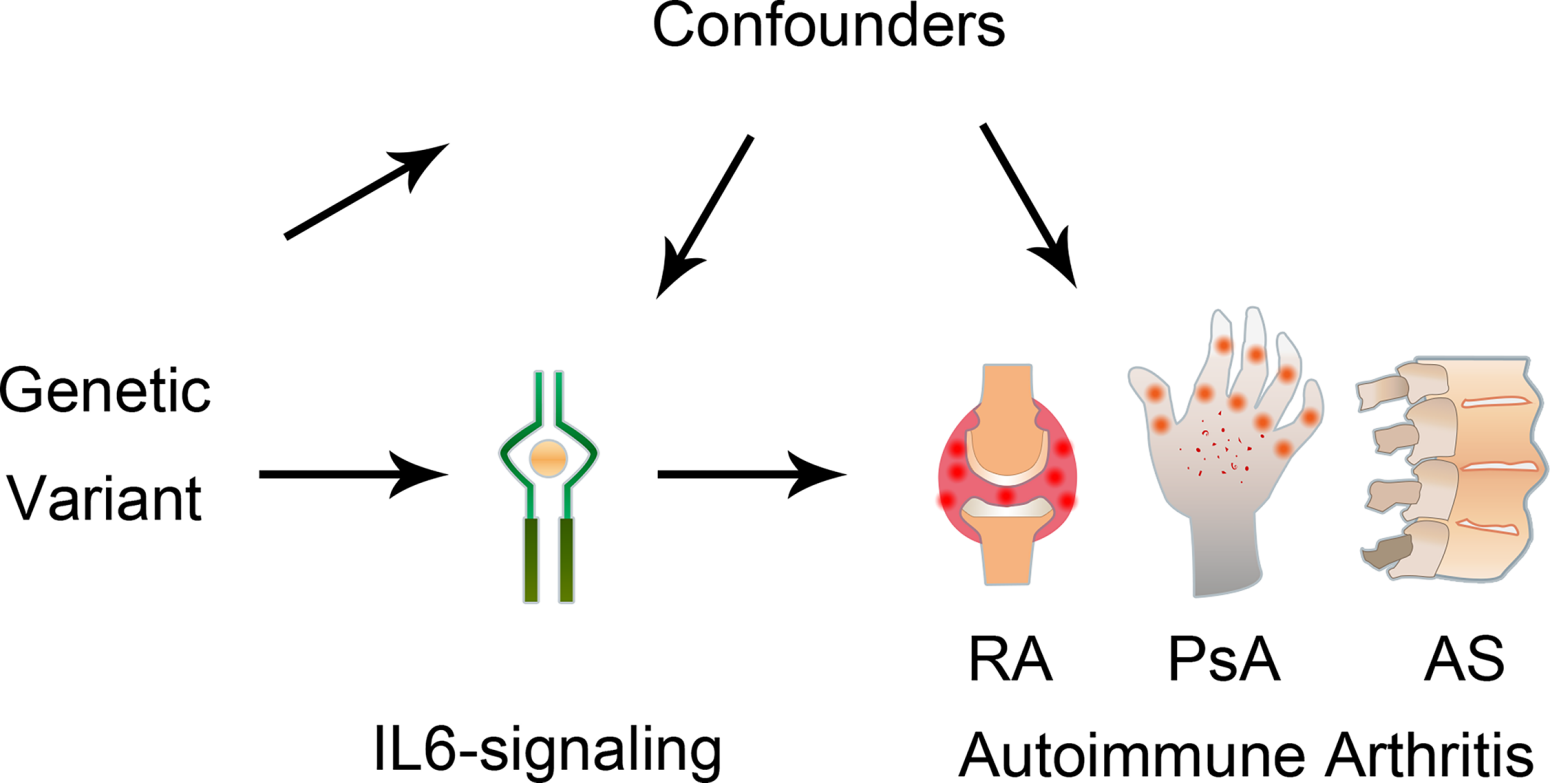
Graphical diagram of the Mendelian randomization analysis between IL6 and risk of autoimmune arthritis. RA, rheumatoid arthritis; AS, ankylosing spondylitis; PsA, psoriatic arthritis.

**Figure 2:**
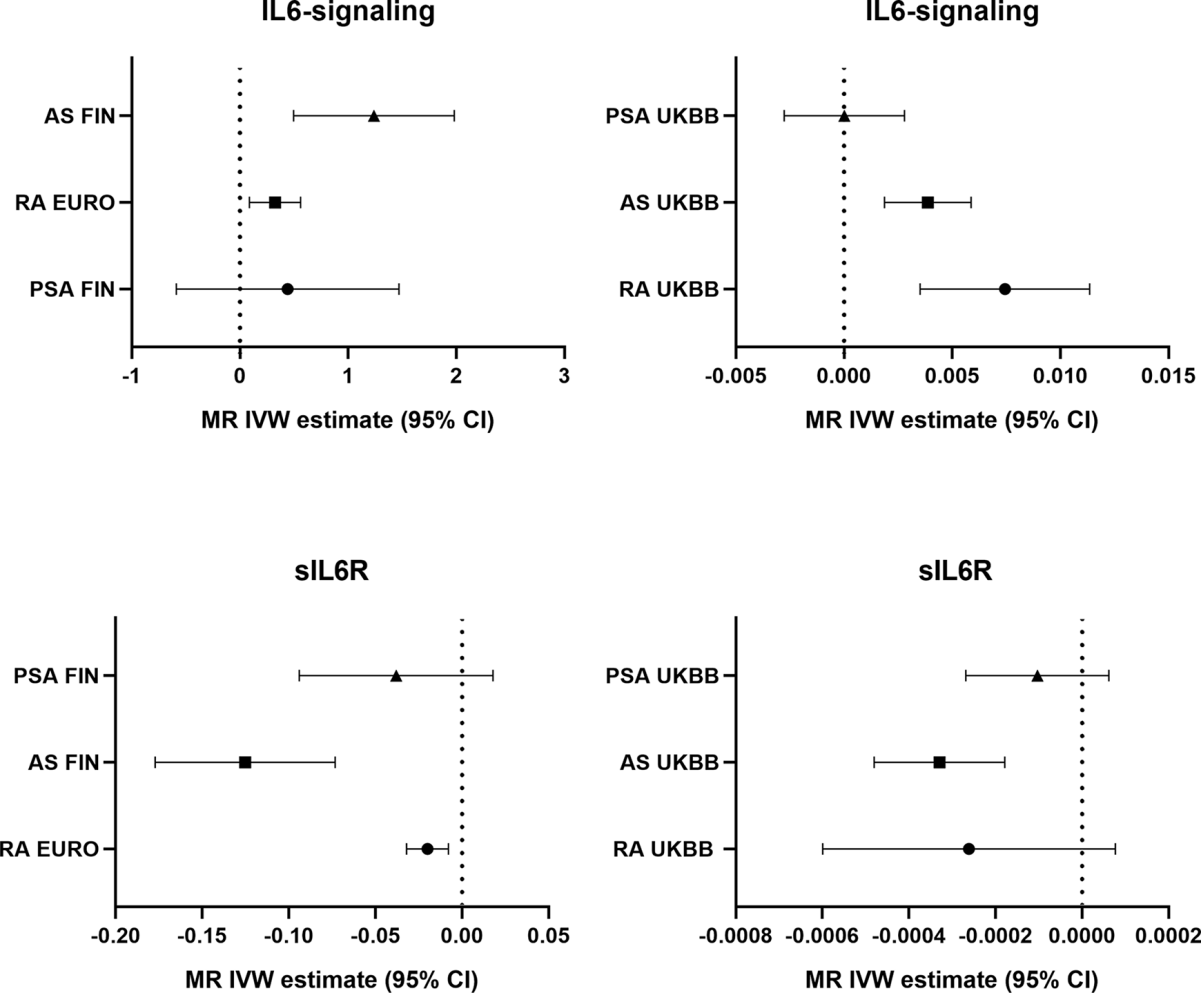
Forest plot of the causal effects of IL6-signaling and sIL6R on autoimmune arthritis.

Potential genetic association between the levels of IL6-signaling, sIL6R, and autoimmune arthritis were detected by using two-sample MR analysis (**Figure 1**). Summarized results of associations are shown in **Table 1**, and a forest plot is shown in **Figure 2**.

**Table 1:** MR estimates of the causal effect of IL6-signaling and sIL6R level on the risk of autoimmune arthritis.

Exposure	MR method	Outcome	Association			Heterogeneity		Egger regression	MR PRESSO
			beta	se	pval	Q	P-value	P-value	P-value
IL6-signaling	ME	RA	0.053156	0.46213	0.914	19.34	**0.0017**	0.5733	**0.015**
	WM	(Euro)	0.327559	0.07645	**2E-05**				
	IVW		0.324962	0.12067	**0.0071**				
	ME	RA	-0.00496	0.0071	0.5234	3.54	0.6174	0.1430	0.09
	WM	(UKBB)	0.005779	0.00241	**0.0163**				
	IVW		0.007435	0.002	**0.0002**				
	ME	AS	1.470001	1.53846	0.3934	5.33	0.3766	0.8838	**0.021**
	WM	(Finngen)	1.434537	0.46354	**0.002**				
	IVW		1.239619	0.37985	**0.0011**				
	ME	AS	0.003159	0.00363	0.433	1.30	0.9352	0.8485	0.237
	WM	(UKBB)	0.003863	0.00122	**0.0015**				
	IVW		0.003868	0.00102	**0.0002**				
	ME	PsA	1.20307	2.09803	0.5971	11.21	**0.0474**	0.7250	0.069
	WM	(Finngen)	0.579845	0.43903	0.1866				
	IVW		0.441713	0.52576	0.4008				
	ME	PsA	0.002501	0.00548	0.6717	14.02	**0.0155**	0.6613	**0.006**
	WM	(UKBB)	0.000954	0.00108	0.3762				
	IVW		1.77E-05	0.00142	0.99				
sIL6R	ME	RA	-0.02508	0.01238	0.0524	50.80	**0.0074**	0.6351	**0.003**
	WM	(Euro)*	-0.02599	0.0064	**5E-05**				
	IVW		-0.01995	0.00615	**0.0012**				
	ME	RA	-0.00021	0.00036	0.5684	48.29	**0.0418**	0.8518	**0.026**
	WM	(UKBB)	-0.00034	0.00021	0.1018				
	IVW		-0.00026	0.00017	0.1283				
	ME	AS	-0.15921	0.05436	**0.0062**	20.85	0.9503	0.4793	**0.035**
	WM	(Finngen)	-0.12799	0.0384	**0.0009**				
	IVW		-0.12524	0.0265	**2E-06**				
	ME	AS	-0.00029	0.00016	0.0697	35.73	0.3414	0.8073	0.085
	WM	(UKBB)	-0.00027	0.0001	**0.0111**				
	IVW		-0.00033	7.6E-05	**2E-05**				
	ME	PsA	-0.07011	0.05904	0.2437	41.86	0.1386	0.5380	0.086
	WM	(Finngen)	-0.04221	0.03518	0.2302				
	IVW		-0.03802	0.02852	0.1825				
	ME	PsA	0.000118	0.00017	0.4816	62.73	**0.0014**	0.1337	**<0.001**
	WM	(UKBB)	-0.00011	8.9E-05	0.2077				
	IVW		-0.0001	8.4E-05	0.2143				

6 IV SNPs were used in IL6-signaling; 34 IV SNPs were used in sIL6R MR analysis. *30 IV SNPs were identified in the Euro RA database. Significant results are in bold. MR, Mendelian randomization; ME, MR-Egger; WM, weighted median; IVW: inverse variance weighted.

### Finding for Upregulated IL6-Signaling and Levels of sIL6R in Autoimmune Arthritis

Casual effects of upregulated IL6-signaling have been found to be positively associated with RA (IVW mode effect=0.325, 95%CI 0.088 to 0.561, p=7.08E-03) and AS (IVW mode effect=1.240, 95%CI 0.495 to 1.980, p=1.1E-03). As the results of MR present, the elevated concentration of sIL6R, which inhibits IL6-signaling, was negatively associated with the risk of RA (IVW mode effect=-0.020, 95%CI -0.0320 to -0.008, p=1.18E-03) and AS (IVW mode effect=-0.125, 95%CI -0.177 to -0.073, p=2.29E-06). Replicated MR analysis confirmed the connection between IL6 and risk of RA (IVW mode effect=0.003, 95%CI 0.007 to 0.011, p=1.98E-04) or AS (IVW mode effect=0.0039, 95%CI 0.0019 to 0.0059, p=1.51E-04). The negative association between sIL6R and AS was validated through replicated MR analysis (IVW mode effect=-0.00033, 95%CI -0.00048 to -0.00018, p=-0.00018). However, no genetic association was found between RA and sIL6R in the replicated MR analysis. Similar with the MR analysis results in PsA, no genetic effects were found between the risk of PsA and IL6-signaling (**Table 1**).

Pleiotropy test using MR-Egger did not denote any pleiotropic SNP in the MR study. However, the horizontal pleiotropy was detected among the MR results by using MRPRESSO (**Table 1**). After removing the pleiotropic SNPs by the MRPRESSO outlier test, the corrected MR estimates are listed in **Table S2**.

Furthermore, we carried out Cochran’s Q statistics test to evaluate the MR results. There was no sign of heterogenetic effects between the genetic effects and risk of AS (**Table 1**). However, heterogeneity was found among the MR estimate concerning the association between IL6 and two other autoimmune arthritis diseases (**Table 1**). Thus, leave-one-out sensitivity analysis was performed to identify the important SNP and the possible source of heterogeneity (**Figure S1**). Rs2228145, a well-reported IL6 receptor genetic variant, was confirmed to strongly influence the effects of IL6-signaling on RA or AS (28) (**Figure S1**). In addition, rs4129267, an IL6R-related SNP in LD with rs2228145(r2 = 1) identified by Ferreira and her colleges (29), was found to make a great contribution to getting RA. On the other hand, rs12059682 had a biased role of IL6 in the onset of PsA (**Figure S1**).

### Gender-Stratified MR Analysis for IL6-Signaling and Levels of sIL6R in Autoimmune Arthritis

**Figure 3:**
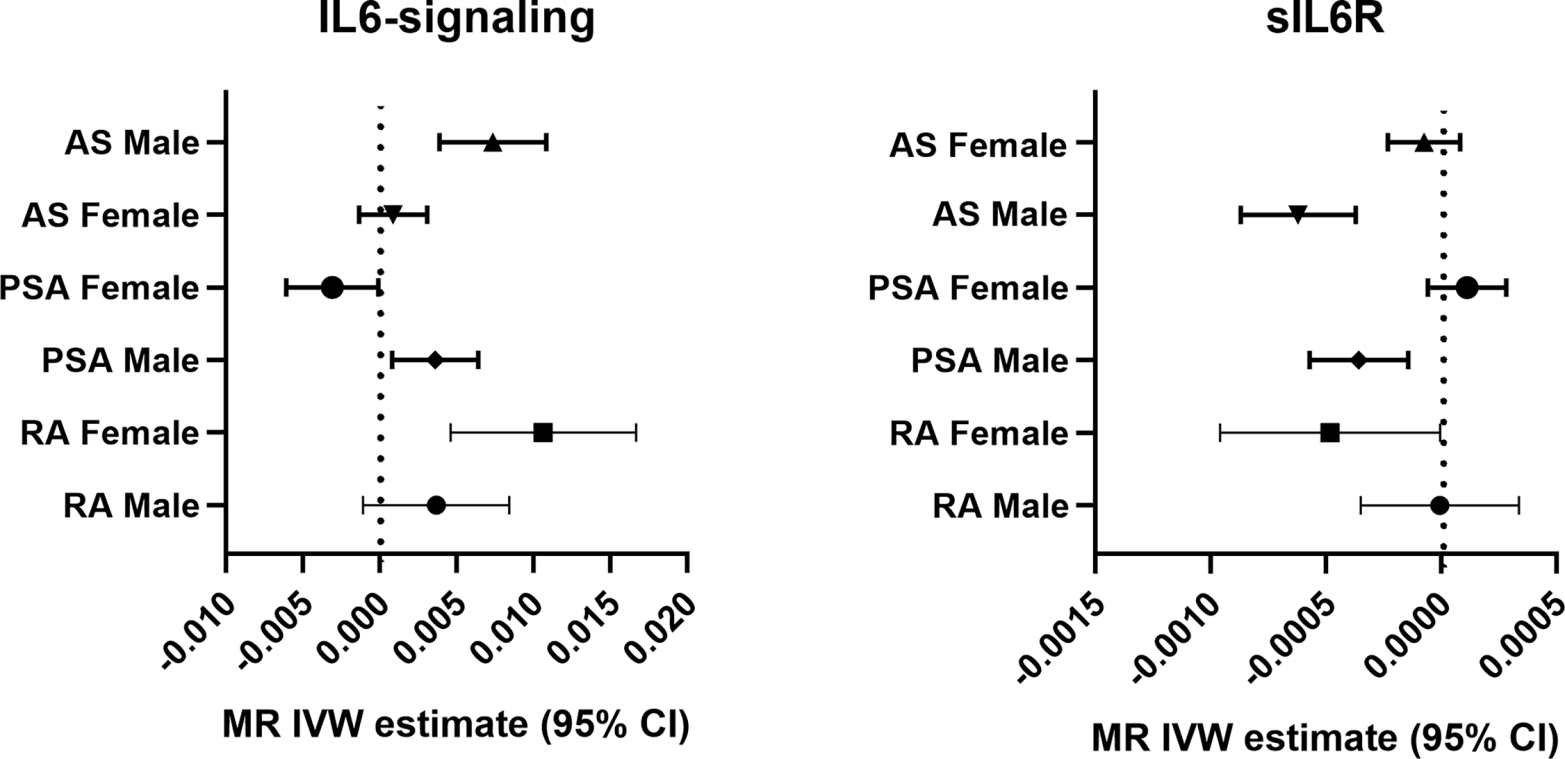
Forest plot of the causal association between IL6-signaling/sIL6R and autoimmune arthritis by sex.

Sexual dimorphic effects on autoimmune arthritis were widely demonstrated previously. In order to further identify the potential heterogeneity in order to further identify among MR analyses. We performed sex-stratified MR analysis to judge whether a sex-specific genetic relationship existed between the IL6 and the risk of autoimmune arthritis (**Figure 3**). The UKBB GWAS datasets were used, which include publicly available sex-specific summary data. We found casual association between IL6 and RA in women rather than in men. Conversely, the male population seemed easier to suffer from AS when exposed to elevating IL6-signaling levels (**Table 2**). Consistent with the casual estimate in AS, men could be at higher risk of getting PsA than women. However, it is intriguing that upregulation of IL6-signaling even negatively associated with the risk of PsA (**Table 2**). Next, as shown by MRPRESSO, the pleiotropy in the MR estimate was found only in PsA. After removing pleiotropic SNP from MR analysis, there was no casual association between IL6 and PSA in the female population (corrected estimate listed in **Table S2**).

**Table 2:** Gender-stratified MR estimates of the causal effect of IL6-signaling and sIL6R level on the risk of autoimmune arthritis.

Exposure	MR method	Outcome	Association			Heterogeneity		Egger regression	MR PRESSO
			beta	se	pval	Q	P-value	P-value	P-value
IL6	ME	RA	-0.00517	0.010933	0.661162	3.002	0.6996	0.206	0.124
-signaling	WM	(Female)	0.008674	0.003873	**0.02512**				
	IVW		0.010639	0.003077	**0.00054**				
	ME	RA	-0.00465	0.008614	0.617867	2.240	0.8150	0.371	0.669
	WM	(Male)	0.00236	0.002809	0.400914				
	IVW		0.003676	0.002425	0.129564				
	ME	AS	-0.00083	0.004016	0.846193	1.855	0.8689	0.682	0.854
	WM	(Female)	0.000379	0.001309	0.771996				
	IVW		0.000869	0.00113	0.441816				
	ME	AS	0.007835	0.006303	0.28172	0.661	0.9850	0.942	0.105
	WM	(Male)	0.007138	0.002134	**0.00082**				
	IVW		0.007363	0.001774	**3.3E-05**				
	ME	PsA	0.002284	0.005417	0.694948	9.320	0.0970	0.360	**0.046**
	WM	(Female)	-0.00168	0.001378	0.222713				
	IVW		-0.00308	0.001534	**0.04443**				
	ME	PsA	0.002806	0.005655	0.64576	6.215	0.2859	0.888	**0.033**
	WM	(Male)	0.003881	0.001518	**0.01056**				
	IVW		0.003621	0.001428	**0.01122**				
sIL6R	ME	RA	-0.00034	0.000501	0.505225	40.382	0.1764	0.740	0.141
	WM	(Female)	-0.00059	0.000318	0.06377				
	IVW		-0.00048	0.000244	**0.04763**				
	ME	RA	-5E-05	0.000352	0.887085	30.849	0.5746	0.882	0.561
	WM	(Male)	-1E-04	0.000245	0.684076				
	IVW		-4.5E-06	0.000174	0.9794				
	ME	AS	-4E-05	0.000164	0.807609	29.348	0.6496	0.807	0.627
	WM	(Female)	-8.9E-06	0.00012	0.940617				
	IVW		-7.6E-05	8.11E-05	0.351547				
	ME	AS	-0.00059	0.000258	**0.02848**	26.696	0.7728	0.896	0.311
	WM	(Male)	-0.00055	0.000186	**0.00307**				
	IVW		-0.00062	0.000127	**1.1E-06**				
	ME	PsA	0.000286	0.000175	0.111997	38.330	0.2404	0.261	0.319
	WM	(Female)	0.00013	0.000112	0.247094				
	IVW		0.000112	8.7E-05	0.197332				
	ME	PsA	-7.9E-05	0.000218	0.720009	46.942	0.0547	0.154	**0.001**
	WM	(Male)	-0.00032	0.000134	**0.01541**				
	IVW		-0.00036	0.00011	**0.00117**				

Significant results are in bold. MR, Mendelian randomization; ME, MR-Egger; WM, weighted median; IVW, inverse variance weighted

Next, we apply the leave-one-out sensitive test to evaluate whether some potential SNP creates bias on the gender effects on the MR estimate. No SNP was discovered to create bias in the genetic effect between IL-6 exposure and RA or AS (**Table 2**). Accordingly, Rs2228145 was also verified as the key SNP in the development of PsA in men (**Figure S2**). Because rs12059682 was identified as the potential bias SNP among overall PsA populations, we found that it posed gender difference in the effect contribution to the onset of PsA (**Figure S2**). When leaving it out, upregulating IL6-signaling was also found to be not associated with the risk of PsA.

To deeply identify the role of rs12059682 in the PsA among different genders, firstly, the genetic variant was mapped as the variation of ADAR (adenosine deaminase acting on RNA). Single-tissue eQTL evaluation was carried out using GTEX. Rs12059682 T>C variation would lead to a higher transcriptional level of ADAR in the adrenal gland, EBV-transformed lymphocytes, lung esophagus mucosa, and fibroblast (**Figure S3** and **Table S3**). Based on Shallev et al.’s work (30), decreased A-to-I RNA editing could lead to the accumulation of (double-stranded RNA) dsRNA, which played an important role in psoriasis pathogenesis. Variation of rs12059682 was found riskier in women. Therefore, we brought out the hypothesis that a lower level of ADAR promoted the development of PsA in females.

**Figure 4:**
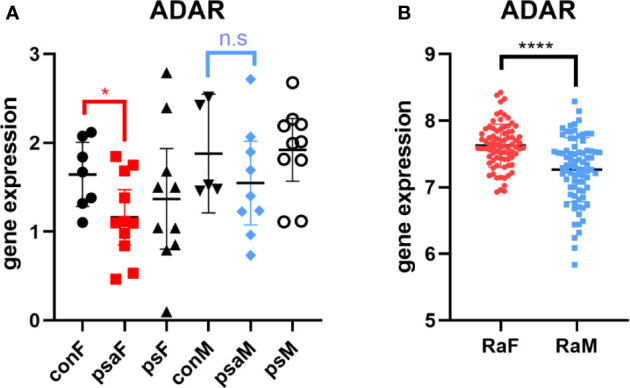
Expression of ADAR in RA or PsA from GEO datasets GSE61281 **(A)** and GSE74143 **(B)**. con, control; psa, psoriatic arthritis; ps, psoriasis; RA, rheumatoid arthritis; F, female; M, male. Data from all bar graphs are presented as mean ± SEM. (*P < 0.05, ****P < 0.0001, n.s, not significant).

Thus, we browsed the GEO database for sex-stratified PsA gene expression profile. As shown by **Figure 4A**, the transcriptional level of ADAR was lower in female PsA patients than healthy donors (GSE61281, p=0.0371) (31), while there was no statistically significant difference between male PsA patients and the control group (p=0.3333). In addition, the ADAR level was found to be correlated with RA (32). The rs12059682 T>C mutation could result in a higher level of ADAR and increased the risk of developing RA based on the GWAS datasets. Moreover, the level of ADAR was found to be higher in female RA patients compared to age-matched male patients (**Figure 4B**, GSE74143) (32).

### Regional Visualization of Autoimmune Arthritis GWAS Datasets for Further Analysis

**Figure 5:**
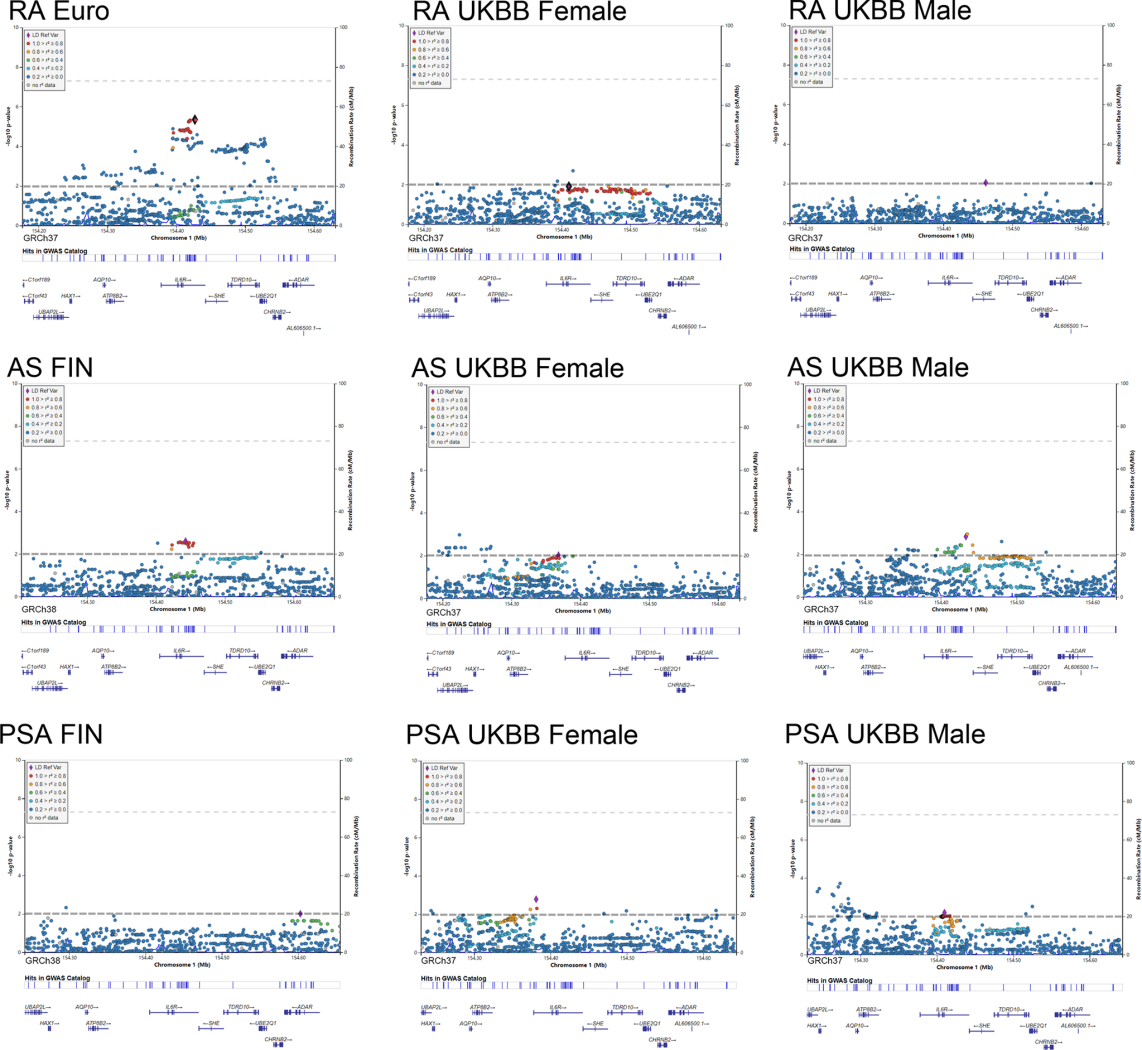
Regional plot of the genetic variants at 1q21.3 and their association with autoimmune arthritis.

As shown in **Figure 5**, LocusZoom plots were displayed based on the IL6R gene region (1q21.3) to present evidence of genetic colocalization between diseases. However, no genome-wide level association signals were found in this region. At first, we browsed the IL6R gene region to identify the top SNPs with a p-value lower than 0.01 (MAF >0.01). If the candidate SNP could not be annotated to the RS number and substituted by proxy SNP, then the SNP in this region with the next lowest p-value was chosen for analysis.

By using the Finngen GWAS dataset for AS, rs4845372 (1:154442920_C/A, P=2.48 × 10^-3) was identified as the potential SNP accounting for IL6’s effects in the onset of AS. After LD evaluation, this genetic variance was found to be in high linkage with rs2228145 (r2 = 0.882). Next, a sex-stratified plot using UKBB GWAS data presented rs6695045 (1:154432957_A/G, P=1.47 × 10^-3) as the top influencer in the IL6R region with the male population, which was also an SNP that was relatively in linkage with rs2228145 (r2 = 0.438). On the other hand, in the female population, none of the candidate SNPs (p<0.01) was detected in the IL6R gene region. When looking for other than the IL6R gene region, there were no other signals having eQTL data or a literature-based relationship with IL6R.

In addition, our regional plot further emphasized rs2228145 (1:154426970_A/C, P=4.50 × 10^-6) as the lead SNP, putting great genetic predisposition to RA (**Figure 5**). As for further sex-specific analysis, no SNP in the IL6R region met our criteria when analyzing male GWAS data. Rather, a weak signal (1:154410686_C/CT p=0.012) was identified in women with RA. The C to T variance of proxy SNP rs55800510 (R2 = 0.9666) was associated with the risk of RA (**Table S3**).

As for the regional plotting result in male PsA patients, we found that rs12730036 (1:154416969_C/T p=9.37 × 10^-3) was in high linkage with rs2228145 (r2 = 0.941). This result denoted consistency with the MR analysis, which resulted in a higher risk of PsA in male by upregulating IL6 signaling. Interestingly, the lowest-p value-polymorphism rs12083537 G>A, which was able to activate the IL6-related pathway, presented to be a protective genetic variance for women against PsA (**Table S3**).

## Discussion

In the present MR study, by applying two sets of genetic IVs, our findings showed corroborating evidence that the overactive IL6 signal pathway led to autoimmune arthritis, especially in RA and AS. Genetic predisposition to a higher IL6 level was also genetically associated with the risk of PsA in the male population. Consistent with a previous study, elevating level of IL6 was found in patients with autoimmune arthritis, which played a great role in the pathogenesis of joint lesions (6, 7, 33). As Rosa and her colleague presented, by using sIL6R genetical IV and PheWAS analysis, the genetically determined sIL6R was negatively associated with the risk of RA (15). We extended the result and complemented the evidence from a regional plot and MR analysis. rs2228145, a genetic variant that accounted for 51% variance of sIL6R levels (34), was identified as the common susceptibility locus for autoimmune arthritis. Additionally, the strategy of targeting the IL6 axis by using novel biological medicine to treat RA had been well validated (35). However, anti-IL6 therapy against AS or PsA was still not introduced clinically.

We observed that sexual differences influenced IL6-intermediate susceptibility to autoimmune arthritis. Sex differences were reported in the level of IL-6 among monocytes (36). Consistently, gender differences in the prevalence of RA were well described based on some large epidemiological studies (37). In the previous study, hormonal factors and sex chromosomes were thought to cause the sex difference in autoimmune disease (38, 39). Here, we present the evidence that IL6 acted differently during the onset of RA, which could be another possible explanation for gender difference in RA (40). Likewise, AS is another typical autoimmune arthritis where the sex ratio is 3:1 (M/F) (41). Our MR analysis confirmed the casual association between IL6 and the development of AS in a gender-dependent manner. However, Gracey and his colleagues found that there was no sex dimorphism in the level of IL-6 among AS patients (42). Additionally, another observational study identified a higher level of IL6 in female patients with syndesmophytes than those without them. No significant change of the IL6 level was found in the pooled populations or male patients with syndesmophytes (43). Here, by using the summary dependent t-test, we found that the IL6 level was higher in male than in female patients without syndesmophytes (3.46 ± 2.86 *vs* 1.47 ± 1.1) (p=0.024). We presumed that the result of the IL6 level was confounded by the progression of diseases. However, the underlying mechanism is still unclear and further study is needed.

As for the onset of PSA, unlike the equal gender ratio observed in epidemiology, we found that a genetically elevating level of IL6 could increase the risk of PsA for men and decrease the risk for women. A cross-sectional analysis reported by Eder et al., who specifically compared the sexual difference in PsA, presented the phenotype that male PsA patients developed more severe axial and joint damage than female (44). Contrarily, some researchers reported that female patients were suffering from RA with more severe joint damages (45, 46). Here we identified a genetic variant rs12059682, located in the ADAR1 gene region, causing heterogeneity in MR analysis. A recent study introduced the role of the IL6R-STAT3-ADAR1 axis in the oncogenicity of multiple myeloma (47). It should be noted that enrichment of ADAR SNP loci was identified in GWAS signals for autoimmune diseases (48). Corresponding to the evidence from the rna-seq dataset, rs12059682 C>T mutation could lead to a lower level of ADAR in the lymphocyte and fibroblast. The decreased level of DNA editing, regulated by the ADAR (49), was correlated with a higher risk of PsA due to the accumulation of dsRNA (30). Furthermore, the impaired post-translational modification of dsRNA and unwinding of dsRNA structures led to adaptive immune activation through producing IFNβ (50). On the other hand, Vlachogiannis et al. observed elevated adenosine-to-inosine RNA editing in the progress of RA (32). Consistently, a higher transcriptional level of ADAR was observed in female RA patients. As Vlachogiannis et al. suggested, more single-stranded RNA (ssRNA) was produced by adenosine-to-inosine RNA editing, which resulted in the binding of ssRNA-binding proteins and increase in the expression of these pro-inflammatory genes (such as HuR). Therefore, the opposite effect of ADAR could be the reason for a different role of IL6 in these two autoimmune arthritis diseases. Elsewise, this SNP could partially account for the sexual difference in RA or PsA susceptibility. However, further sex-stratified GWAS research and pathological studies should be carried out to validate these hypotheses and undermine the potential mechanism.

This MR study was performed by using two sets of IL6-related IV and multiple GWAS datasets, which aimed to ensure the consistency of the results. However, there were still some limitations. Firstly, the sex-stratified MR analysis was mainly based on the UKBB GWAS data. Some genetic variants were different from Finland and UK populations, which was caused by “population bottlenecks” (51). Gender-stratified GWAS datasets from Finland should be collected to replicate the findings, although the allele frequency of major SNP was similar between the two ethnic groups. Moreover, our study identified rs12059682 as the potential source of bias. Other GWAS focused on the level of ADRA editing that should be performed to identify the casual association between ADRA and autoimmune arthritis. Finally, however, the genetic pleiotropy was rectified by the MRPRESSO in the pooled or sex-stratified MR under a sensitive test. Other potential pathway for the associations cannot be fully ruled out.

To summarize, we identified the genetic association between IL6-signaling and onset of autoimmune arthritis. These results further validated IL6 inhibition as the therapeutic strategies for RA. Rather, the genetic role of IL6 in a sex-dependent manner was discovered in the development of male AS, male PsA, and female RA. Those who have started randomized controlled trials of IL6 inhibition on autoimmune arthritis should take gender differences into account. In the end, the hypothesis that ADAR reduces the effectiveness of the IL6 inhibitor in PsA populations, supported by the statistical findings, should be further investigated through pathophysiological studies.

## Data Availability Statement

The original contributions presented in the study are included in the article/**Supplementary Material**. Further inquiries can be directed to the corresponding authors.

## Author Contributions

SY, JH, and WW designed this study. Study conduct: JH and WW. Data collection: ZQ, CL, XJ, and JW. Data analysis: CF, YZ, XJ, and GZ. Drafting the manuscript: JH, ZQ, and XJ. Revising the manuscript content: JM, CJ, YS, and CZ. Approving the final version of the manuscript: all authors. SY takes responsibility for the integrity of the data. All authors contributed to the article and approved the submitted version.

## Funding

This research was supported by the National Natural Science Foundation of China under Grant Nos. 81772360 and 81800782, Zhejiang Province Medical and Health Project No. 2020391395, and Yinzhou District Agriculture and Social Development Science and Technology Project No. 2017YZQ104.

## Conflict of Interest

The authors declare that the research was conducted in the absence of any commercial or financial relationships that could be construed as a potential conflict of interest.

## Publisher’s Note

All claims expressed in this article are solely those of the authors and do not necessarily represent those of their affiliated organizations, or those of the publisher, the editors and the reviewers. Any product that may be evaluated in this article, or claim that may be made by its manufacturer, is not guaranteed or endorsed by the publisher.

## Supplementary Material

Supplementary Figure S1 Forest plots and “leave-one-out” plot for MR analyses of the causal effect of IL6 on autoimmune arthritis in pooled population.

Supplementary Figure S2 Forest plots and “leave-one-out” plot for MR analyses of the causal effect of IL6 on autoimmune arthritis in female or male population.

Supplementary Figure S3 Violin plot and multiple tissues expression level of rs12059682.
