# Supplementary figures and images for: Genetic Associations Between IL-6 and the Development of Autoimmune Arthritis Are Gender-Specific
Source: Front Immunol. 2021 Sep 3;12:707617. doi: 10.3389/fimmu.2021.707617 (PMC8447937; doi:10.3389/fimmu.2021.707617)

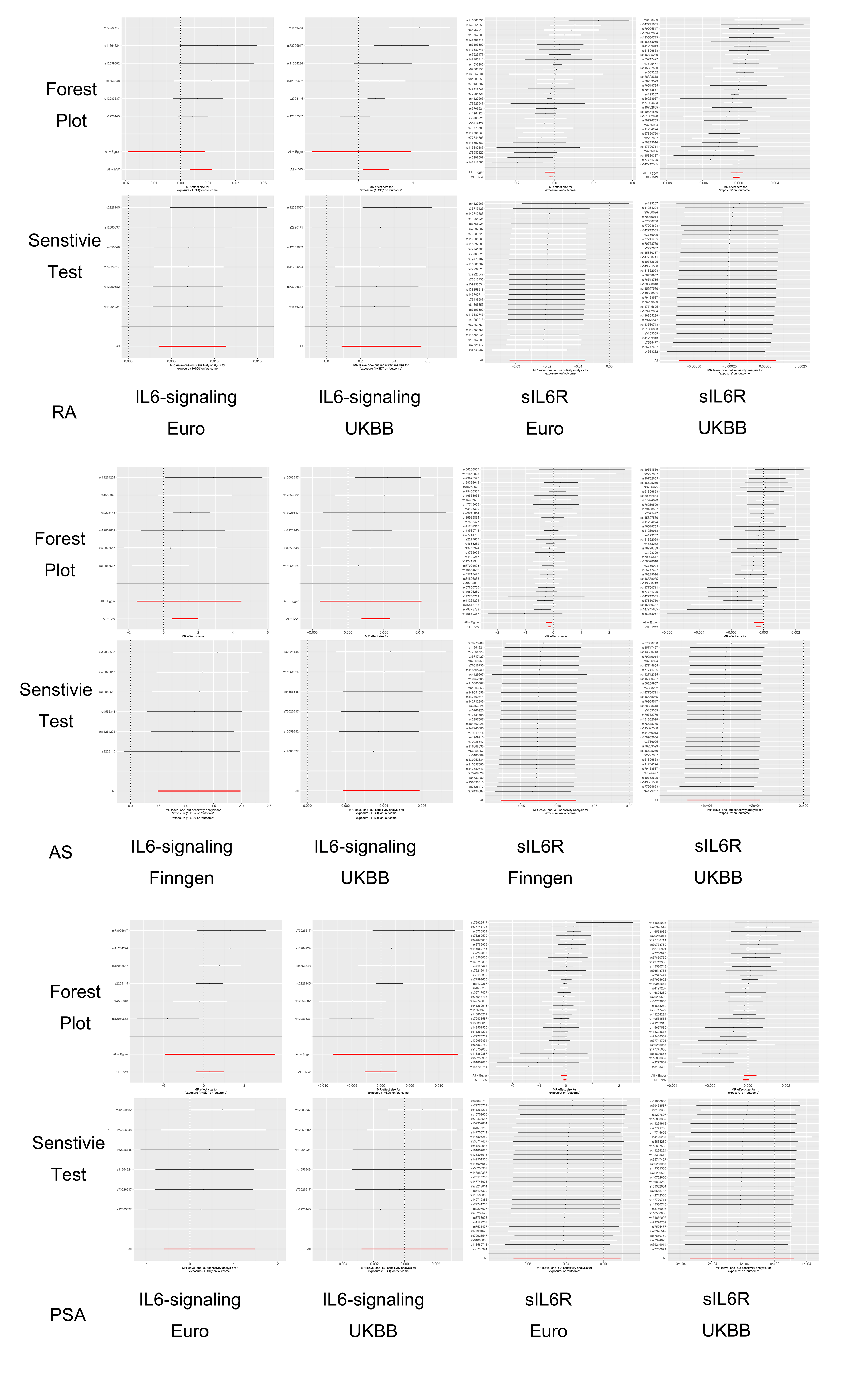

Supplement: Supplementary Figure S1 — Forest plots and “leave-one-out” plot for MR analyses of the causal effect of IL6 on autoimmune arthritis in pooled population. [file Image_1.png]

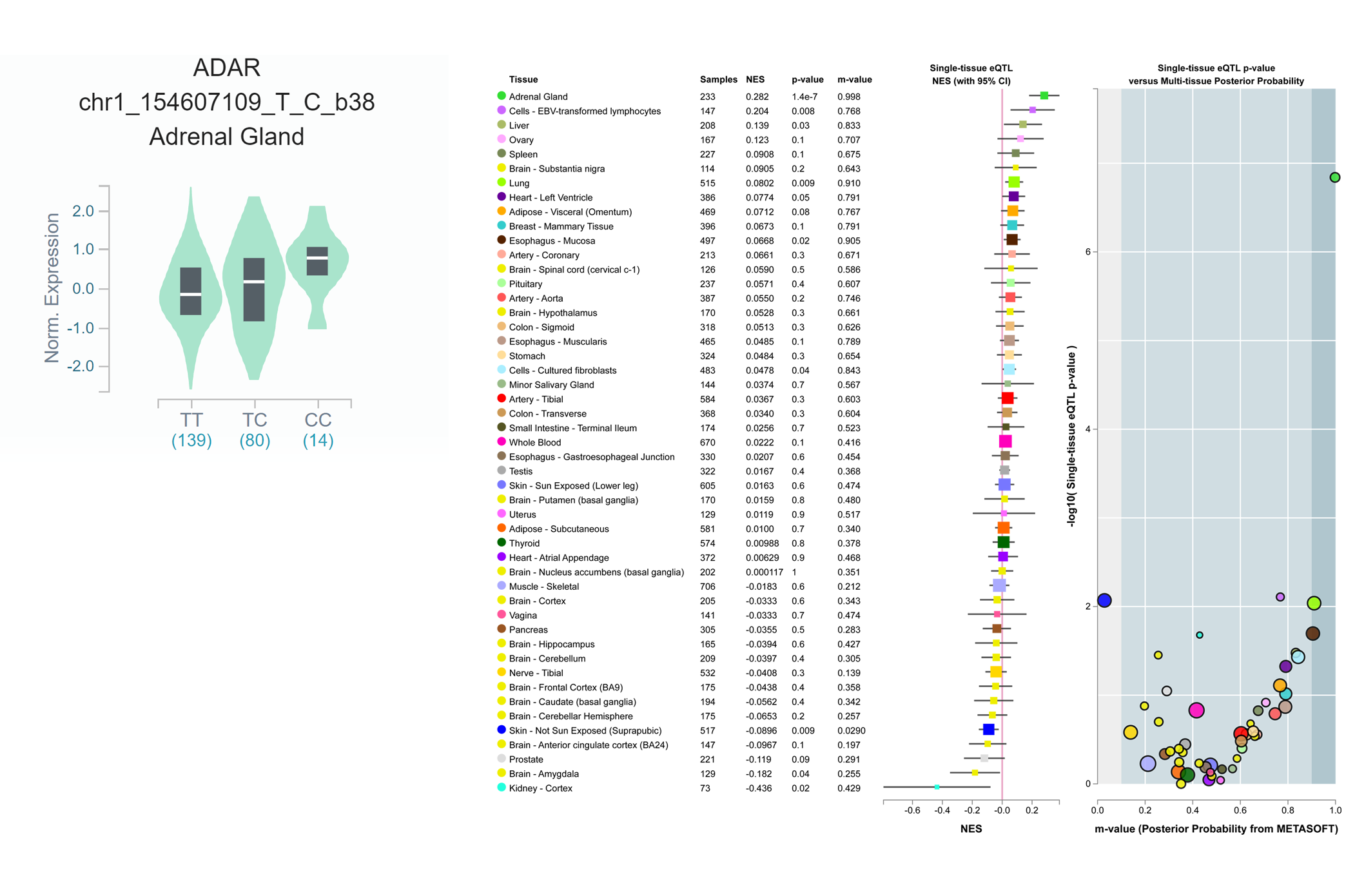

Supplement: Supplementary Figure S3 — Violin plot and multiple tissues expression level of rs12059682. [file Image_3.tif]
