# Supplementary material for: Genetic Associations Between IL-6 and the Development of Autoimmune Arthritis Are Gender-Specific
Source: Front Immunol. 2021 Sep 3;12:707617. doi: 10.3389/fimmu.2021.707617 (PMC8447937; doi:10.3389/fimmu.2021.707617)
Supplement: Supplementary file 4 [file Table_1.docx]

| **TableS1. instrumental variant for MR anaylsis** | | | | |  |  |  |
| --- | --- | --- | --- | --- | --- | --- | --- |
| **Exposue** | **SNP** | **Effect allele** | **Reference allele** | **Beta** | **SE** | **P** | **nearest gene** |
| IL6-signaling |  |  |  |  |  |  |  |
|  | rs73026617 | T | C | 0.0474 | 0.0068 | 3.16E-12 | IL6R |
|  | rs12083537 | A | G | 0.0643 | 0.0053 | 7.14E-34 | IL6R |
|  | rs4556348 | T | C | 0.0541 | 0.0067 | 6.77E-16 | IL6R |
|  | rs2228145 | A | C | 0.0899 | 0.0042 | 1.21E-101 | IL6R |
|  | rs11264224 | A | C | 0.0465 | 0.0057 | 3.41E-16 | ADAR |
|  | rs12059682 | T | C | -0.0441 | 0.0049 | 2.26E-19 | ADAR |
| sIL6R |  |  |  |  |  |  |  |
|  | rs61806853 | T | C | -0.4957 | 0.0573 | 5.01 x 10-18 | TPM3 |
|  | rs181862028 | A | T | -0.4133 | 0.1042 | 7.24 x 10-5 | HAX1 |
|  | rs3103309 | T | C | 0.16 | 0.0261 | 8.51 x 10-10 | HAX1 |
|  | rs2297607 | A | G | 0.1756 | 0.0291 | 1.66 x 10-9 | ATP8B2 |
|  | rs56258967 | T | C | 0.4718 | 0.1151 | 4.17 x 10-5 | ATP8B2 |
|  | rs116568035 | A | G | -0.3112 | 0.0696 | 7.76 x 10-6 | ATP8B2 |
|  | rs79438587 | T | C | 0.405 | 0.0338 | 4.07 x 10-33 | ATP8B2 |
|  | rs35717427 | A | G | 0.5238 | 0.036 | 5.62 x 10-48 | IL6R |
|  | rs7525477 | A | G | -0.3502 | 0.0261 | 4.79 x 10-41 | IL6R |
|  | rs79778789 | A | G | -0.7852 | 0.0887 | 8.32 x 10-19 | IL6R |
|  | rs79219014 | T | G | 0.7582 | 0.0767 | 4.57 x 10-23 | IL6R |
|  | rs139952834 | T | C | -0.6506 | 0.1072 | 1.29 x 10-9 | IL6R |
|  | rs113580743 | A | G | -0.5141 | 0.0605 | 1.95 x 10-17 | IL6R |
|  | rs4129267 | T | C | 1.1148 | 0.0157 | 7.41 x 10-1101 | IL6R |
|  | rs142712385 | A | T | -0.2782 | 0.0534 | 1.91 x 10-7 | IL6R |
|  | rs77741705 | C | G | 0.5205 | 0.0941 | 3.16 x 10-8 | IL6R |
|  | rs79925547 | T | C | 0.752 | 0.1155 | 7.41 x 10-11 | IL6R |
|  | rs147700711 | T | G | -0.4972 | 0.119 | 2.95 x 10-5 | IL6R |
|  | rs76518735 | A | C | 0.5646 | 0.0924 | 1.00 x 10-9 | SHE |
|  | rs41269913 | T | C | 0.6983 | 0.0599 | 1.95 x 10-31 | SHE |
|  | rs77994623 | T | C | -0.6184 | 0.0307 | 2.75 x 10-90 | TDRD10 |
|  | rs4633282 | T | C | 0.6124 | 0.0286 | 6.31 x 10-102 | TDRD10 |
|  | rs116805289 | A | C | 0.6267 | 0.0822 | 2.51 x 10-14 | TDRD10 |
|  | rs76289529 | T | C | 0.6656 | 0.0678 | 9.77 x 10-23 | TDRD10 |
|  | rs115697580 | A | G | -0.4684 | 0.0967 | 1.26 x 10-6 | TDRD10 |
|  | rs149551556 | T | C | -0.6651 | 0.0947 | 2.19 x 10-12 | UBE2Q1 |
|  | rs67860750 | C | G | 0.3985 | 0.0353 | 1.41 x 10-29 | CHRNB2 |
|  | rs138398618 | A | G | -0.5044 | 0.1027 | 9.12 x 10-7 | CHRNB2 |
|  | rs3766925 | A | T | 0.184 | 0.0294 | 3.89 x 10-10 | ADAR |
|  | rs11264224 | A | C | -0.4576 | 0.0336 | 3.89 x 10-42 | ADAR |
|  | rs3766924 | T | C | -0.3863 | 0.03 | 7.94 x 10-38 | ADAR |
|  | rs115880387 | A | G | -0.6209 | 0.1388 | 7.59 x 10-6 | ADAR |
|  | rs147745605 | T | C | 0.5012 | 0.1034 | 1.26 x 10-6 | ADAR |
|  | rs10752605 | A | G | -0.3349 | 0.0374 | 3.63 x 10-19 | ADAR |
