# Supplementary material for: Genetic Associations Between IL-6 and the Development of Autoimmune Arthritis Are Gender-Specific
Source: Front Immunol. 2021 Sep 3;12:707617. doi: 10.3389/fimmu.2021.707617 (PMC8447937; doi:10.3389/fimmu.2021.707617)
Supplement: Supplementary file 5 [file Table_2.docx]

| **TableS2. Corrected MR estimates between IL6-signaling/sIL6R level and risk of autoimmune arthritis** | | | | | | | | | |
| --- | --- | --- | --- | --- | --- | --- | --- | --- | --- |
| **Exposure** | **MR method** | **Outcome** | **No. of** | **Association** |  |  | **Heterogeneity** | | **MRPRESSO** |
|  |  |  | **SNPs** | **beta** | **se** | **pval** | **Q** | **P-value** | **P-value** |
| Pooled population | |  |  |  |  |  |  |  |  |
| IL6-signaling | ME | RA(Euro) | 4 | 0.1190974 | 0.22283187 | 0.6464757 | 3.050681 | 0.383877 | 0.296 |
|  | WM |  |  | 0.3462106 | 0.0754436 | **4.4538E-06** |  |  |  |
|  | IVW |  |  | 0.3767528 | 0.07096918 | **1.1042E-07** |  |  |  |
|  | ME | AS(Finngen) | 5 | 1.6131904 | 1.72522 | 0.41873398 | 4.990569 | 0.2882665 | 0.164 |
|  | WM |  |  | 1.3350984 | 0.4850775 | **0.0059171** |  |  |  |
|  | IVW |  |  | 1.1619587 | 0.4367861 | **0.00780835** |  |  |  |
|  | ME | PsA(UKBB) | 4 | 0.02599211 | 0.029057351 | 0.4654419 | 11.2079 | 0.0474102 | 0.059 |
|  | WM |  |  | 0.00184553 | 0.002032496 | 0.3638717 |  |  |  |
|  | IVW |  |  | 0.00067156 | 0.002147771 | 0.754525 |  |  |  |
| sIL6R | ME | RA(Euro) | 28 | -0.02684585 | 0.010339297 | **0.0152923** | 31.86734 | 0.2370331 | 0.296 |
|  | WM |  |  | -0.02780658 | 0.006037491 | **4.1118E-06** |  |  |  |
|  | IVW |  |  | -0.02654531 | 0.005456113 | **1.1432E-06** |  |  |  |
|  | ME | RA(UKBB) | 33 | -0.00027291 | 0.000345743 | 0.43590212 | 43.46557 | 0.08503216 | 0.052 |
|  | WM |  |  | -0.00033829 | 0.000212382 | 0.1112009 |  |  |  |
|  | IVW |  |  | -0.00023956 | 0.000167674 | 0.15308185 |  |  |  |
|  | ME | PsA(UKBB) | 32 | -0.00017619 | 0.000164364 | 0.2922949 | 41.26547 | 0.10290635 | 0.07 |
|  | WM |  |  | -0.00011184 | 8.52764E-05 | 0.1896851 |  |  |  |
|  | IVW |  |  | -6.8682E-05 | 7.06354E-05 | 0.3308764 |  |  |  |
| Female population | |  |  |  |  |  |  |  |  |
| IL6-signaling | ME | PsA(UKBB) | 5 | 0.00280358 | 0.004022753 | 0.5359969 | 4.569737 | 0.3343584 | 0.361 |
|  | WM |  |  | -0.00104647 | 0.001417433 | 0.4603422 |  |  |  |
|  | IVW |  |  | -0.00191398 | 0.001331447 | 0.1505698 |  |  |  |
| Male population | |  |  |  |  |  |  |  |  |
| IL6-signaling | ME | PsA(UKBB) | 5 | 0.00468388 | 0.00544006 | 0.45255547 | 4.0917 | 0.3937379 | 0.105 |
|  | WM |  |  | 0.00355225 | 0.001561522 | **0.02291417** |  |  |  |
|  | IVW |  |  | 0.00317313 | 0.001332253 | **0.0172292** |  |  |  |
| sIL6R | ME | PsA(UKBB) | 32 | -0.00028367 | 2.03E-04 | 0.17251282 | 30.96575 | 0.4679392 | 0.095 |
|  | WM |  |  | -0.00032117 | 1.33E-04 | **0.01613683** |  |  |  |
|  | IVW |  |  | -0.00030216 | 9.31E-05 | **0.00116561** |  |  |  |
| ME: MR-Egger, WM: Weighted median , IVW:Inverse variance Weighted | | | | | |  |  |  |  |
