# Supplementary material for: Genetic Associations Between IL-6 and the Development of Autoimmune Arthritis Are Gender-Specific
Source: Front Immunol. 2021 Sep 3;12:707617. doi: 10.3389/fimmu.2021.707617 (PMC8447937; doi:10.3389/fimmu.2021.707617)
Supplement: Supplementary file 6 [file Table_3.docx]

| **TableS3.SNPs whose effects measured by eQTL analysis or GWAS Catalog in this study** | | | | | | | |  |  |  |
| --- | --- | --- | --- | --- | --- | --- | --- | --- | --- | --- |
| **SNP** | **Gene** | **Outcome** | **Effect allele** | **Reference allele** | **beta** | **se** | **pval** | **eQTL site/ GWAS Catalog Trait** | **Effect** | **pval** |
| rs55800510 | IL6R | RA UKBB Female | T | C | 0.0012153 | 0.00052215 | 0.0199402 | Artery - Aorta | 0.195 | 2.20E-05 |
|  |  |  |  |  |  |  |  | Cells - EBV-transformed lymphocytes | 0.484 | 0.006 |
| rs6695045 | IL6R | AS UKBB Male | G | A | -0.00071748 | 0.00022556 | 0.00146833 | Artery - Tibial | -0.155 | 1.30E-10 |
| rs12730036 | IL6R | PsA UKBB Male | T | C | -0.0004168 | 0.00016042 | 0.00937301 | Artery - Tibial | -0.204 | 9.9E-19 |
| rs12083537 | IL6R | PsA UKBB Female | G | A | 0.00052788 | 0.0001676 | 0.00163512 | C-reactive protein measurement | 0.064 unit decrease | 1 x 10-33 |
| rs12059682 | ADAR | PsA UKBB Male | C | T | 1.7518E-05 | 0.00019557 | 0.928628 | Adrenal Gland | 0.282 | 1.40E-07 |
|  |  | PsA UKBB Female | C | T | -0.00041577 | 0.00017158 | 0.0153862 | Cells - EBV-transformed lymphocytes | 0.204 | 0.008 |
